# The Molecular Basis and Therapeutic Potential of* Let-7* MicroRNAs against Colorectal Cancer

**DOI:** 10.1155/2018/5769591

**Published:** 2018-06-19

**Authors:** Rei Mizuno, Kenji Kawada, Yoshiharu Sakai

**Affiliations:** Department of Surgery, Graduate School of Medicine, Kyoto University, Kyoto, Japan

## Abstract

Although a number of studies have revealed the underlying mechanisms which regulate the development of colorectal cancer (CRC), we have not completely overcome this disease yet. Accumulating evidence has shown that the posttranscriptional regulation by the noncoding RNAs such as microRNAs plays an important role in the development or progression of CRC. Among a number of microRNAs, the* let-7* microRNA family that was first discovered in* C. elegans* and conserved from worms to humans has been linked with the development of many types of cancers including CRC. The expression level of* let-7* microRNAs is temporally low during the normal developmental processes, while elevated in the differentiated tissues. The* let-7* microRNAs regulate the cell proliferation, cell cycle, apoptosis, metabolism, and stemness. In CRC, expressions of* let-7* microRNAs have been reported to be reduced, and so* let-7* microRNAs are considered to be a tumor suppressor. In this review, we discuss the mechanisms regulating the* let-7* microRNA expression and the downstream targets of* let-7* in the context of intestinal tumorigenesis. The application of* let-7* mimics is also highlighted as a novel therapeutic agent.

## 1. Introduction

Colorectal cancer (CRC) is one of the most common causes of cancer deaths worldwide [1–3], and the number of CRC patients is increasing more and more [4]. Many studies have revealed the molecular mechanisms of the colonic tumorigenesis and have proposed various models such as the adenoma-carcinoma sequence (i.e., multistep carcinogenesis) and colitis-associated carcinogenesis observed in inflammatory bowel diseases (IBD). However, we have not yet completely understood the molecular basis of CRC tumorigenesis. One of the reasons which makes our understanding difficult is that not only genetic changes, such as mutation/deletion of oncogenes or tumor suppressor genes, but also the epigenetic modification or posttranscriptional regulation by RNA binding proteins and microRNAs is involved in the colonic tumorigenesis.

The* let-7* microRNAs are noncoding RNAs which consist of ~22 nucleotides [5]. They are first discovered in* C. elegans* as a critical regulator of developmental process [6, 7].* Let-7* microRNAs have been experimentally confirmed to be conserved in a wide range of species including humans [8]. As the* let-7 *family, 9 members are identified so far:* let-7a, let-7b, let-7c, let-7d, let-7e, let-7f, let-7g, let-7i, and miR-98*. The mature* let-7* negatively regulates the expression of target mRNAs at a posttranslational level via imperfect base pairing to the 3'-UTR of their target mRNAs [9]. During normal developmental process, the expression levels of* let-7* microRNAs in stem and progenitor cells are maintained low. As progenitor cells differentiate,* let-7* expression is increasing [10].

*Let-7* microRNAs are downregulated in various types of cancers such as hepatocellular carcinoma (HCC), gastric adenocarcinoma, pancreatic cancer, ovarian cancer, prostate cancer, Burkitt lymphoma, renal cell carcinoma, breast cancer, and melanoma [11]. In the context of CRC,* let-7* microRNAs are also downregulated, which affects the posttranscriptional regulation of target mRNAs resulting in the colonic tumorigenesis and progression.

In this review, we discuss the molecular mechanisms by which* let-7* microRNAs regulate the colonic tumorigenesis, tumor progression, and chemotherapy resistance by a posttranscriptional regulation and also highlight the possibility of the application of* let-7* targeting therapy against CRC.

## 2. Biogenesis of* Let-7* MicroRNAs (Figure 1)

Mature* let-7 *microRNAs are synthesized through the multiple processing by microprocessors [12].* Let-7* microRNAs are initially transcribed in long transcripts called primary* let-7* (pri-*let-7*), which are processed in the nucleus by Drosha and Pasha to pre-*let-7* with hairpin structures of about 70 nucleotides. Next, pre-*let-7* are exported to the cytoplasm by exportin-5, where they are subsequently processed by the enzyme Dicer resulting in the mature* let-7* [12]*. Let-7* is known to be negatively regulated by the RNA binding proteins, LIN28A or LIN28B. LIN28A or LIN28B has two RNA binding domains, cold shock domain (CSD) and two Cys-Cys-His-Cys- (CCHC-) type zinc finger domains (ZFDs) [13–16], which bind to the conserved GGAG motif in the terminal loop of pri-*let-7* or pre-*let-7*, respectively, resulting in the inhibition of the maturation of* let-7* [17, 18]. The binding of LIN28A or LIN28B to either pri-*let-7* or pre-*let-7* inhibits the processing of* let-7* precursors by Drosha and Dicer [19]. Upon binding to pre-*let-7*, LIN28A or LIN28B recruits TUT4/TUT7, which causes oligouridylation at the 3′ terminal of pre-*let-7* [20–22]. Under normal conditions, Dicer recognizes the two nucleotides at the 3′ terminal via its PAZ domain; however, oligouridylation elongates the 3′ terminal resulting in the resistance to Dicer cleavage. Oligouridylated pre-*let-7* can also be degenerated by the 3′-5′ exonuclease Dis312 [23, 24]. Thus, LIN28A or LIN28B not only inhibits the biogenesis of* let-7* family miRNAs, but also induces their degradation. LIN28A and LIN28B are the negative regulators of* let-7* microRNAs, and so the expression level of LIN28A is high in the developmental process, while that of LIN28B is low in the adult tissues [10]. LIN28B has two isoforms: LIN28B-long and LIN28B-short isoforms. Both isoforms preserve the binding ability to pre*-let-7*. However, LIN28B-short isoform lacks the inhibitory ability against the processing by microprocessors. Namely, LIN28B-short isoform functions as a competitor against the LIN28B-long isoform in the context of* let-7* maturation [25].

## 3. *Let-7* and Colon Cancer

Regarding the LIN28AB/*let-7* axis, the downregulation of* let-7* or upregulation of either LIN28A or LIN28B has been reported to be related to the worse prognosis in CRC patients. Stage I/II CRC patients with high expression of LIN28B exhibit poorer prognosis compared to those with no LIN28B expression [26]. King et al. reported that the expression level of LIN28B was inversely correlated to that of mature* let-7a* in human CRC [27]. Tu et al. examined the expression of LIN28A or LIN28B in the cohort of almost 600 CRC patients and found that LIN28A or LIN28B was highly expressed in 38 % of CRC patients [28]. Importantly, the overall survival was lower in the patients with high expression of LIN28A or LIN28B than in those with low expression of LIN28A or LIN28B. Madison et al. demonstrated that intestinal tumors were spontaneously developed in the transgenic mice with intestinal-specific LIN28B expression (*Villin-Lin28b*), which suggests that LIN28B functions as an oncogene [29].* Let-7b* and* let-7c* can escape the downregulation by LIN28B in mice. Therefore, they crossed these mice with* let-7b* and* let-7c* knockout mice (*let-7*^*IEC-KO*^) and found that the additional downregulation of* let-7b* and* let-7c* resulted in the increase of tumor incidence [30]. These results suggest that* let-7b* or* let-7c* plays an important role in the intestinal tumorigenesis in mice.

## 4. *Let-7* Target mRNAs in Intestine

As* let-7* target mRNAs in the intestinal epithelium, Madison et al. revealed that* HMGA1, HMGA2, IGF2BP2, HIF3A, ARID3A, and E2F5* were the most highly induced targets of* let-7* microRNAs in the context of intestinal homeostasis [31]. Among these target molecules, the protein expression levels of HMGA1, HMGA2, ARID3A, and HIF3A were dramatically increased in the intestinal tumors. They also demonstrated that HMGA2 plays an important role in the downstream of* let-7* in the context of colonic tumorigenesis. They found that the forced expression of HMGA2 could increase the colony forming capacity in intestinal organoids and that the haploinsufficiency of* HMGA2* significantly decreased the tumor incidence in* villin-Lin28b* mice. HMGA2 is a member of the High Mobility Group A class of proteins which bind to AT-rich DNA stretches and modulate gene expression by introducing structural alterations in the chromatin landscape [32]. HMGA2-deficiency impaired the growth in mice, whereas the transgenic expression of HMGA2 variants enhanced the formation of benign tumors, indicating that HMGA2 confers a growth advantage and thus promotes tumorigenesis [33]. In fact, overexpression of HMGA2 was reported to promote metastasis and impact the survival of CRC patients [34]. RNA binding protein IGF2BP1 (IMP1) is also considered as a* let-7* target molecule and highly expressed during developmental process, and prominent upregulation and/or de novo synthesis are observed in various tumors [33, 35]. Hamilton et al. demonstrated that enforced expression of IMP1 in SW480 cells could increase the growth of xenografts [36], which indicates that IMP1 plays an important role in the formation or dissemination of colon cancer.

## 5. Functions of* Let-7* in the Colonic Tumorigenesis

### 5.1. Cell Cycle, Proliferation, and Apoptosis

The downregulation of* let-7* promotes cell proliferation through the activation of a variety of cellular proliferation signaling and cell cycle signaling pathways. For instance,* let-7* negatively regulates RAS expression through direct binding, which could downregulate MAPK pathway and PI3K/AKT pathway [11, 37].* Let-7 *also directly downregulates some oncoproteins such as MYC, HMGA2, and IGF1 [38–41] that are known as critical regulators for the growth of CRC.

The downregulation of* let-7* upregulates some cell cycle regulators such as cyclin A2, cyclin D1/2, CDK6, CDC34, CDC25A, Aurora A and B kinases, CDK8, PLAGL2, and TRIM71, which results in the activation of cell cycle [11, 42].* Let-7* also inhibits cell proliferation by regulating transcriptional factors such as STAT3, E2F5, E2F6, CBFB, PLAGL2, SOX9, GZF1, YAP1, GTF2I, and ARID3A [42, 43].* Let-7* microRNAs also suppress cell proliferation by regulating Wnt signaling. The inhibition of Wnt signaling by* let-7* is reported in various types of cancers [44–47]. In the context of intestinal tumorigenesis, Tu et al. demonstrated that overexpressed LIN28 could accelerate the growth of intestinal tumors in* Apc*^*Min /+*^ mice and that this tumor-promoting effect was* let-7 *dependent [28].

The relationship between* let-7* microRNAs and apoptosis in CRC is still controversial. Geng et al. reported that* let-7* inhibited apoptosis by decreasing the expression of Fas through the direct inhibition against* Fas* mRNA in HT29 cells [48]. They demonstrated that* let-7* inhibition increased Fas expression and the sensitivity to the FAS-related apoptosis. Conversely, Zhang et al. demonstrated that forced expression of* let-7c* promoted apoptosis in CRC cell lines at least by targeting BCL2L1 [49]. Mongroo et al. showed that the loss of IMP1, a* let-7* target, promoted caspase- and lamin-mediated cell death through CYFIP2 by the cross-talk with KRAS in SW480 cells [50]. Another* let-7* target in intestine, HMGA2, was reported to inhibit apoptosis [51]. The effect of* let-7* microRNAs on apoptosis might be context-dependent and so further investigation will be needed.

### 5.2. *Let-7* and Intestinal Stem Cells

Intestinal stem cell marker LGR5 is known to be upregulated in human colon cancers and sporadic adenomas [67]. The intestinal stem cell markers such as LGR5, ASCL2, SMOC2, Msi1, and Tert are also known to be increased in colitis-associated carcinogenesis [68]. Madison et al. examined the expression levels of* let-7* microRNAs and intestinal stem cell markers (e.g.,* LGR5, EPHB2,* and* ASCL2*) in human CRC tissues and normal adjacent tissues.* Let-7a *and* let-7b* were significantly downregulated in CRC specimens, while intestinal stem cell markers were significantly upregulated, which suggests that depletion of* let-7a* and* let-7b* may contribute to a stem cell phenotype in CRC [31]. They also demonstrated that tumors from* Villin-Lin28b/let-7*^*IEC-KO*^ mice exhibited a significant upregulation of stem cell markers including Bmi1, Lrig1, Olfm4, ASCL2, Prom1, LGR5, Msi1, and SOX9, suggesting an expansion of CRC and +4 stem cell-like compartments. They evaluated the coexpression of* let-7* target mRNAs and stem cell markers in mouse samples, and found that* HMGA1* and* HMGA2* were intensely correlated with stem cell markers.* HMGA2* expression was also correlated with* LGR5* expression in human CRC samples [31].

### 5.3. Tumor Progression and* Let-7 *MicroRNAs in CRC

The downregulation of* let-7* promotes migration and invasion of normal intestinal epithelial cells and CRC cells [27]. Xenografts of the DLD-1 cells in which* let-7* expression was decreased by LIN28B overexpression developed lung and liver metastases in a mouse model, which suggests that LIN28B/*let-7* axis could affect the metastasis in CRC [26]. Some* let-7* target oncogenes such as RAS and MYC might contribute to the tumor progression [69, 70]. HMGA2, one of the* let-7* target molecules in intestine, is reported to promote epithelial-to-mesenchymal transition (EMT) through the induction of transcription factor Slug [71]. Forced expression of another* let-7* target IMP1 in CRC cell line promoted the growth of xenograft tumors and dissemination into the blood in a mouse model. IMP1 overexpression decreased the expression of E-cadherin, suggesting that IMP1 contributes to the tumor progression through the loss of epithelial identity [36].

### 5.4. *Let-7* and Chemoresistance

The downregulation of* let-7 *has been reported to contribute to the acquisition of resistance against chemotherapy in many types of cancers such as breast cancer [72], esophageal cancer [43], acute myeloid leukemia [73], pancreatic cancer [74], ovarian cancer [75], and HCC [76]. Although only a single paper reported that the high* let-7g* expression was significantly associated with the chemoresistance against S-1 in CRC patients [77], most of the studies have shown that the downregulation of* let-7* contributes to the chemoresistance in the context of CRC. One of the underlying mechanisms by which the downregulated* let-7* contributes to the chemoresistance is that the decreased* let-7* induces the expression of DNA excision repair protein, excision repair cross-complementing group 1 (ERCC1), which contributes to the resistance against cisplatin or 5-FU [25].

*Let-7* microRNAs play an important role in regulating the response to antiepidermal growth factor receptor (EGFR) therapies in CRC patients. Cappuzzo et al. demonstrated that the KRAS wild-type metastatic CRC patients with high* let-7c* exhibited better response to EGFR monoclonal antibodies [78]. Although anti-EGFR treatment is not effective in CRC patients with mutated KRAS, Ruzzo et al. reported that the upregulation of* let-7a* may rescue the sensitivity of anti-EGFR therapies in such CRC patients [79]. A functional variant of a* let-7* complementary site (LCS6) in the KRAS 3′UTR mRNA might be a useful biomarker to predict the sensitivity of anti-EGFR therapies in patients with metastatic CRC, and further prospective confirmation is needed [80].

## 6. *Let-7 *MicroRNAs as a Therapeutic Target

As discussed above, accumulating evidence has indicated that* let-7* microRNAs function as a tumor suppressor in CRC. Therefore, gene replacement therapy which attempts to introduce the analogous* let-7* molecules could be effective. Recent advances in genetic engineering have enabled us to make the microRNA mimics, artificial structures of RNA duplexes, that are identical to the mature microRNA sequence. An microRNA mimic is designed to have the function of the endogenous microRNA, attempting to restore its loss of function as a tumor suppressor [81]. However, systemic administration of microRNAs mimics might induce unexpected side effects because microRNAs including* let-7* can be functional not only in cancer cells but also in normal cells (i.e., development, cell proliferation, apoptosis, cell cycle control, differentiation, migration, and metabolism [82, 83]). Therefore, alternative delivery systems functionalized with miRNA using nanoparticles have recently gained intense attention [53] and have been investigated* in vitro/in vivo* and also in clinical trials. Different types of microRNA mimics have been used in the medical field as therapeutic agents loaded on the surface of nanoparticles (Table 1).* Let-7* mimics have been proved to have therapeutic efficacy in mouse models of lung and prostate cancers [55, 84]. These delivery systems have been tested on several animal models, and some disadvantages regarding toxicity, immune, and inflammatory responses were observed [85].

For clinical application, gene replacement therapy with microRNA mimics met many obstacles: the instability of therapeutic molecules, nonspecific inflammation, controlled release of therapeutic molecules, specificity and efficiency of the delivery systems [53]. Advanced delivery strategies are still needed.

## 7. Conclusions

Accumulating evidence has showed that* let-7* microRNAs function as tumor suppressor through the posttranscriptional regulation of target mRNAs in CRC.* Let-7* microRNAs regulate various biological events such as cell proliferation, cell cycle, migration, progression, stem cell biology, metabolism, and chemoresistance. Gene replacement therapy with* let-7* mimics which attempts to restore the tumor suppressive function of* let-7* is tested and therapeutic efficacy has been proved in both* in vitro* experiments and* in vivo* animal models. However, many obstacles still remain to be overcome for the safe clinical application. Advanced drug delivery strategies are required.

## Figures and Tables

**Figure 1:**
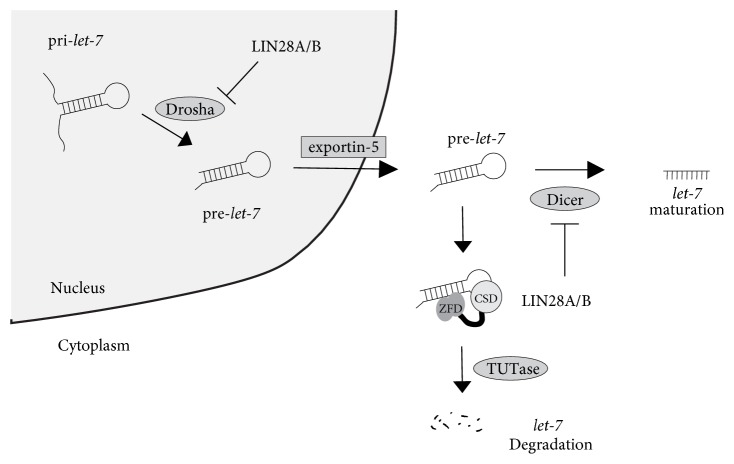
**A schematic diagram of the* let-7* biogenesis**. Pri-*let-7* microRNA precursors are processed in the nucleus by Drosha to pre-*let-7*. Pre-*let-7* microRNA precursors are exported to the cytoplasm by exportin-5, where they are subsequently processed by Dicer resulting in the mature* let-7*. RNA binding protein LIN28A or LIN28B binds to either pri-*let-7* or pre-*let-7 *using RNA binding domains, cold shock domain (CSD), and two zinc finger domains (ZFDs) and blocks the processing. Upon binding to pre-*let-7*, LIN28A or LIN28B recruits TUTase, which causes oligouridylation resulting in the degradation of pre-*let-7*.

**Table 1:** MicroRNA replacement therapy with mimics.

**microRNA**	**Cancer**
microRNA-29	Lung [52]
microRNA-31	Neuroblastoma [53]
microRNA-34a	Neuroblastoma [54], Lung [55–57], HCC [58], Pancreas [59], Lymphoma [60], Prostate [61], Multiple Myeloma [62]
microRNA-113b	Lung [63]
microRNA-143	CRC [64], Pancreas [59]
microRNA-145	Pancreas [59], Lung [65]
microRNA-365	CRC [66]
*LET-7*	Lung [55]

CRC: colorectal cancer; * *HCC: hepatocellular carcinoma.
